# Supplementary material for: The perceptions of different professionals on school absenteeism and the role of school health care: A focus group study conducted in Finland
Source: PLoS One. 2022 Feb 28;17(2):e0264259. doi: 10.1371/journal.pone.0264259 (PMC8884500; doi:10.1371/journal.pone.0264259)
Supplement: S1 Appendix — (DOCX) [file pone.0264259.s001.docx]

# **Appendix**

**S1 Protocol. Focus group discussion guide.**

1. What kind of damages does school absenteeism cause?
2. How many absences is a lot?
3. What kind of absences should one worry about? How many absences should one become worried about?
4. What kind of reasons cause absences?
5. Besides the reason given for the absence, what other factors influence the situation?
6. What is an excused absence?
7. When do absences become problematic?
8. Whose responsibility is it to intervene in absenteeism?
9. Does one intervene in absenteeism? What hinders interventions?
10. What would be a good method in intervening in absenteeism?
11. How could absenteeism be prevented?
12. Could the school doctor participate in absenteeism interventions? What qualifications should the doctor have to be able to intervene in absenteeism? How could the doctor intervene in absenteeism? Should absentees be referred to the school doctor?
13. Could the school doctor participate in absenteeism prevention? How could the school doctor participate in absenteeism prevention? What qualifications should the doctor have in order to participate in absenteeism prevention?
