# Supplementary material for: The perceptions of different professionals on school absenteeism and the role of school health care: A focus group study conducted in Finland
Source: PLoS One. 2022 Feb 28;17(2):e0264259. doi: 10.1371/journal.pone.0264259 (PMC8884500; doi:10.1371/journal.pone.0264259)
Supplement: S1 Data — (DOCX) [file pone.0264259.s002.docx]

# **Additional information on reasons for absenteeism derived from the ten focus groups**

## STUDENT-RELATED REASONS

“*Quite often, in my opinion, there really are quite serious problems behind [absenteeism]. For instance, perhaps in addition to that, there might be somatic absences and psychiatric indisposition, somatization, and so forth.”* *(school doctor, FG5)*

The most commonly named somatic symptoms were headache, including migraine, and abdominal pain. Pain symptoms were said to produce absences with discernible patterns: they often occur on Mondays and Fridays, biweekly, or on exam days. Participants recognized the somatization component of these symptoms and discussed whether these symptoms exacerbate themselves since absences cause stress. The school doctors contemplated whether the school environment could be organized to allow the student to continue the school day after the migraine has subsided instead of going home for the rest of the day.

“*The absences in our school are mainly caused by abdominal pains and migraines, and they cause hundreds of hours of absences, and then how to tackle them—that is truly quite a problem.”* *(principle, FG6)*

Learning difficulties (LD), both diagnosed and undiagnosed, were another cause for frequent absences on exam days. They lower self-esteem by making school seem difficult, despite the various supportive systems within the school environment. LD were considered a risk factor that should be marked to the electronic student monitoring system so that the system would alert the parents/guardians when the student is skipping classes where LD is of particular importance. The heritability of LD was also discussed and the participants were concerned about the ability of the family to support the student sufficiently.

*“Or then there is some hidden, like, learning difficulty, which might not manifest until middle school, so that in elementary school they still manage but then in junior high school some learning related challenge or difficulty is discovered.” (social worker, FG8)*

Anxiety and depression were the most commonly named mental health problems. Both were viewed as new phenomena relevant to our time and were thought disconcerting because they were difficult to identify. Anxiety was described as an umbrella term that could be the result of numerous factors related to school life. If left untreated, the situation could progress to a point where the student would be thoroughly unable to leave home, another new phenomenon. Sometimes this inability was due to the student’s severe psychiatric illness, at other times it was the result of a cascade where, during a long period of time, different stressors have accumulated, alluring the student to stay at home in an attempt to avoid stress. A facilitator in this process, according to some participants, was the fact that there were no real consequences for this behavior. The parents/guardians were partly culpable to this behavior: they could have a lax attitude towards routines like attending school, or they could lack authority.

*“[...] they start having these absences, and then the student somehow starts getting into this new rhythm where they don’t come [to school] anymore, and then it’s easier and easier, of course, to stay home and not come here at all.”(school social worker, FG2)*

The role of friends in relation to absenteeism was twofold: absenteeism could be the consequence of loneliness or having the wrong kind of friends. The latter might introduce the student to substance use which also causes absences. Alcohol and cannabis were the most commonly named intoxicants, although their role in absenteeism was unclear and perhaps underestimated, said the participants.

*“It is very hard to get a grip on it when there is an unknown amount of absences that are certainly due to the fact that they drink so much alcohol or use cannabis.” (principle, FG6)*

Modern technology was mentioned in multiple contexts. Friends could utilize technology to lure others out of a lesson or it could make staying at home more entertaining. It could also be used for gaming which was said to affect the normal daily routine, thus resulting in absences. In this study, gaming was mentioned separately from other hobbies which were an example of an acceptable reason for absences.

*“But still, for some incredible reason, the school is such an interesting place that they come here. And then it leads to the fact that—since there are cellphones—[absenteeism] is infectious. And then they send [each other] messages saying ‘come out to the lobby’ or ‘come out to the hallway.’ And then, all of a sudden, we have this standard outflux of ‘I’m going to the bathroom’ or then it has evolved to the point where a person, a student, just stands up and disappears.” (special education teacher, FG10)*

Especially in two schools many students participated in competitive sports. These students were deemed to have caring parents and a consistent way of life that supports their school performance. Therefore, absenteeism due to hobbies was considered acceptable.

*“Then these (almost) competitive athletes are their own group as they have caring parents but they go on trips and so on. These are viewed kindly because often they are otherwise so organized for they are capable of training so much so often school work progresses alongside (the training). They might be absent a lot and you don’t need to react to it.” (school psychologist, FG10)*

The issue of truancy and unexcused absences were also discussed. The participants thought that special education students were rarely truant. The electronic student management system causes a delay in identifying truancy since it facilitates finding an acceptable reasons for the absences. Furthermore, lack of school motivation was viewed as either the root cause or a consequence of absenteeism. School motivation was also discussed on a larger scale where the student lacks plans for the future and is thus unable to understand the importance of school. Absenteeism was also viewed as an indicator of the student’s general attitude towards school.

*“Often those who have unauthorized absences, like, it has been observed that they also have other problems, behavioral issues, and they come to school because of friends and then they still don’t come to class.” (headmaster, FG10)*

## FAMILY-RELATED REASONS

*“Then there are new Finns, immigrants, who might perhaps have the problem that they haven’t really understood what kind of opportunity this Finnish primary and lower secondary education really is.” (principle, FG2)*

In most groups, the attitude of the parent/guardian towards school was said to cause absenteeism. They may be poorly educated themselves and lack appreciation for being academic and thus are unable to recognize the problem with absenteeism. They might try to hide the student’s truancy. Generally, the participants thought that parents/guardians rarely admit that the student was truant.

*“… a few of these asocial, careless families where they don’t monitor a whole lot whether the child attends school or not.” (school doctor, FG5)*

Absenteeism was often seen as a result of inadequate parenting skills. In these situations more than one child of the same family stayed at home. Parents/guardians may take the role of a friend rather than a parent/guardian toward the student, leaving the student responsible for their own education. These parents/guardians also have difficulties in setting boundaries. If the parent/guardian is, for example, unwilling to limit the time that the student spends on their cellphone, waking up in the morning would be difficult. Insufficient parenting skills could result in the lack of a daily routine which could also cause absenteeism. Families with parenting problems were thought to need help from different parties, such as health care and child protective services.

*“Of course a youth attending junior high school also has their own responsibility for their school attendance but, somehow, it also shows what kind of attitude the guardian has, so it also has an impact if the guardian is not terribly interested in how they are doing at school; the youth quite easily thinks ‘oh well,’ and then their motivation kind of dies off.” (social worker, FG8)*

If the parent/guardian stays at home either because of health reasons or unemployment, it could influence the student in multiple ways. It could affect the ability of the parent/guardian to support the student. The student may start to display similar symptoms as the parent/guardian or dread leaving the parent/guardian alone. Sometimes the student may stay at home simply to accompany the parent/guardian. Whichever the mechanism, the participants thought that a parent/guardian at home will increase the likelihood of the student staying at home as well.

“*In some cases parents are noticed to have psychiatric problems, so that the parent is so sick that they can’t help or support [the student] with [absenteeism].”* *(social worker, FG9)*

The participants were also concerned about the ability of the parent/guardian to assess the health of the student, for example whether the headache or abdominal pain warrants staying at home. The student may have been dishonest previously which obscures the assessment of the contemporary situation. Some parents/guardians allow absences even for the mildest of symptoms, finding it hard to send the student to school when in discomfort, although they are aware of accumulated absences.

*“So I do understand that guardians, too, sometimes have real difficulties discerning whether the child should stay home or not. Many guardians are themselves concerned about the amount of absences. But then few dare to somehow force [their child] to school, or it’s kind of difficult to force [them] to go, if the youth is strongly of the opinion that they can’t go, even though the symptoms might externally be hard to see or notice for anyone—even the school nurse—whether they have a headache or stomachache.” (school psychologist, FG6)*

Other aspects of family life were also associated with absenteeism. Travel raised mixed feelings. Since it is a form of excused absenteeism, it was deemed unnecessary to intervene in. Nevertheless, it was thought that students should attend school regularly and only grave health reasons, not a wish to sunbathe, warrant an absence. Religion was another family-related reason why education might be undervalued, thus causing absences.

*“Increasingly, students travel around the globe; that is one impacting factor.” (school nurse, FG3)*

## SCHOOL-RELATED REASONS

Bullying was the most common school-related issue mentioned, although its role in absenteeism was unclear. Some groups named it a common pretext for absenteeism although the actual reason was something else. Other groups suspected bullying to be more common than the professionals realized because the student may be embarrassed to admit being bullied. Participants also claimed that schools were unable to effectively intervene in bullying, thus exacerbating absenteeism. Also student-teacher relationships may influence attendance. Similar to friends, teachers could be either a pull or a push factor: students might fear losing the sanctuary of the classroom, causing absenteeism as graduation approaches, or an incompetent teacher might repel students from coming to class. Absenteeism may increase attention from the teacher which may cause anxiety in the student and thus result in more absences.

*“If there are unpleasant students or just one unpleasant student in the class, [a student] might drop out, already just for that. [...] Somebody badmouths [them], in a way. Naturally the person experiences, or somebody might experience it as bullying, even though it might not even be meant like that, but yes, maybe bullying is the right word.” (guidance counselor, FG7)*

The school ethos was perceived to cause absenteeism on some level. In middle school, teachers change according to subject and students change classrooms between lessons. This may either be too demanding for some students or they may exploit this system, slipping out of school between classes. Different teaching methods used in different subjects may also be hard for the unaccomplished students. Another aspect of school culture discussed was the electronic student management system. The system makes it possible for either the parent or even the student to mask the true reason for the absences. This complicates the identifications of situations that should be intervened in.

*“There are also cases where the students themselves have had the guardian’s login details to [the electronic student management system], and this way they have signed off on [the absences].” (teacher, FG1)*
