# Supplementary material for: The perceptions of different professionals on school absenteeism and the role of school health care: A focus group study conducted in Finland
Source: PLoS One. 2022 Feb 28;17(2):e0264259. doi: 10.1371/journal.pone.0264259 (PMC8884500; doi:10.1371/journal.pone.0264259)
Supplement: Attachment — Submitted filename: Review Number PONE-D-21-17857.docx [file pone.0264259.s003.docx]

The perceptions of different professionals on school absenteeism and the role of school health care. A focus group study.

**Number PONE-D-21-17857**

This is an interesting and well designed qualitative research. Its aim is to assess the role of school health services in decreasing absenteeism among adolescent pupils 13 to 15 years in Finland. The potential limit of the paper is that the results may not apply to other countries, but this problem is counter-balanced by the fact that the authors provide a thorough description of the context of education, schooling and school health in their country. The paper should be revised and specify how the issue of absenteeism has been operationalized: it is different to skip school once or twice in a year for a flu or a broken leg, or to repeatedly miss periods and days for vague reasons or for reasons that are even not provided. Also, the authors rightly mention the issue of “drop-outs”, that is youngsters who do not appear anymore for long periods or definitively, but do not tackle this issue specifically.

The introduction and the methods sections should thus be modified accordingly. It would be as well useful to know what the content is of the yearly nurses’ check-ups; same applies to the doctors’ check-ups.

The analysis of the data is carefully described and meets the standards of a qualitative research.

I have no comments on the results.

The discussion is well presented. It would be useful to comment on the lessons learned for readers from outside Finland regarding the role of school health services. Also, I miss a few remarks on what the authors plan to do, as they point out that there is room for improvement in this area. I think, but this is a personal opinion, that many school health services spend a lot of time on screening procedures whose effectiveness is not necessarily evidence-based. They may thus devote more time to the support given to vulnerable pupils and health promotion. But the authors don’t need to comment on this issue.
